# Effect of propofol and sevoflurane on the inflammatory response of patients undergoing craniotomy

**DOI:** 10.1186/s12871-016-0182-5

**Published:** 2016-03-22

**Authors:** Jasmina Markovic-Bozic, Blaz Karpe, Iztok Potocnik, Ales Jerin, Andrej Vranic, Vesna Novak-Jankovic

**Affiliations:** 1 Department of Anaesthesiology and Intensive Therapy, University Medical Centre Ljubljana, Zaloska 7, Ljubljana, SI-1000 Slovenia; 2 Faculty of Natural Science and Engineering, University of Ljubljana, Ljubljana, Slovenia; 3 Institute of Clinical Chemistry and Biochemistry, University Medical Centre Ljubljana, Ljubljana, Slovenia; 4 Department of Neurosurgery, University Medical Centre Ljubljana, Ljubljana, Slovenia; 5 Service de Neurochirurgie, Fondation Ophtalmologique Adolphe de Rothschild, Paris, France

**Keywords:** Craniotomy, Propofol, Sevoflurane, Interleukin 6, Interleukin 8, Interleukin 10

## Abstract

**Background:**

The purpose of this randomised, single-centre study was to prospectively investigate the impact of anaesthetic techniques for craniotomy on the release of cytokines IL-6, IL-8, IL-10, and to determine whether intravenous anaesthesia compared to inhalational anaesthesia attenuates the inflammatory response.

**Methods:**

The study enroled 40 patients undergoing craniotomy, allocated into two equal groups to receive either sevoflurane (*n* = 20) or propofol (*n* = 20) in conjunction with remifentanil and rocuronium. The lungs were ventilated mechanically to maintain normocapnia. Remifentanil infusion was adjusted according to the degree of surgical manipulation and increased when mean arterial pressure and the heart rate increased by more than 30 % from baseline. The depth of anaesthesia was adjusted to maintain a bispectral index (BIS) of 40–60. Invasive haemodynamic monitoring was used. Serum levels of IL-6, IL-8 and IL-10 were measured before surgery and anaesthesia, during tumour removal, at the end of surgery, and at 24 and 48 h after surgery. Postoperative complications (pain, vomiting, changes in blood pressure, infection and pulmonary, cardiovascular and neurological events) were monitored during the first 15 days after surgery.

**Results:**

Compared with patients anaesthetised with sevoflurane, patients who received propofol had higher levels of IL-10 (*p* = 0.0001) and lower IL-6/IL-10 concentration ratio during and at the end of surgery (*p* = 0.0001). Both groups showed only a minor response of IL- 8 during and at the end of the surgery (*p* = 0.57).

**Conclusions:**

Patients who received propofol had higher levels of IL-10 during surgery. Neither sevoflurane nor propofol had any significant impact on the occurrence of postoperative complications. Our findings should incite future studies to prove a potential medically important anti-inflammatory role of propofol in neuroanaesthesia.

**Clinical trial registration:**

Identified as NCT02229201 at www.clinicaltrials.gov

## Background

Anaesthetic technique for craniotomy has to reduce stress response to pain during intubation and surgical manipulation. Emergence from anaesthesia has to be rapid and smooth allowing early postoperative neurological evaluation. Short-acting opioid remifentanil is commonly used for neurosurgical procedures since it allows perfect titration of the analgesic effect to various noxious stimulation intensities, along with rapid recovery and early neurological evaluation [1–5]. The combination of remifentanil and either propofol or sevoflurane has proved a useful anaesthesia technique in elective neurosurgery [6, 7]. A recent multicentre study revealed no difference in early recovery between three groups of patients given total intravenous anaesthesia (TIVA) with either propofol-remifentanil, sevoflurane-remifentanil or sevoflurane-fentanyl. Either technique provided optimal surgical conditions [8].

Anaesthesia and surgery modulate complex immune responses in patients undergoing major surgery [9]. There is a delicate balance between the release of proinflammatory (IL-6, IL-8) and anti-inflammatory (IL-10) cytokines. The cell-mediated immune response can increase the rate of postoperative complications, such as infection, compromised wound healing, cognitive impairment, and cancer progression [9, 10]. An exaggerated proinflammatory response, such as a systemic inflammatory response (SIRS) may lead to haemodynamic decompensation and multi-organ failure (MOF) [9–12]. Many studies have shown that volatile anaesthetics reduce systemic and local inflammatory response during major surgery [11–14]. Animal studies have demonstrated that volatile anaesthetics can induce neuroinflammation, which leads to the decline of cognitive function in rodents and possibly in humans [15, 16]. Thus, we hypothesized that intravenous anaesthesia compared to inhalational anaesthesia attenuates inflammatory response.

It is very important to prevent brain oedema and provide optimal cerebral perfusion and oxygenation during neurosurgical procedures [1]. Optimal neuroprotective strategies include appropriate patient positioning, management of systemic and cerebral haemodynamics, maintenance of fluid, electrolyte and coagulation balance, and postoperative prevention and treatment of pain and postoperative nausea and vomiting [1].

Our aim was to investigate the effect of anaesthetic technique for craniotomy on the release of cytokines IL-6, IL-8 and IL-10, and to determine whether intravenous anaesthesia compared to inhalational anaesthesia attenuates the inflammatory response.

## Methods

This prospective, randomised, double-blind single-centre study was conducted at the Department of Anaesthesiology and Surgical Intensive Therapy and at the Department of Neurosurgery between 2010 and 2014. It was carried out in close cooperation with the Institute of Clinical Chemistry and Biochemistry, University Medical Centre Ljubljana (trial registry: NCT02229201 at www.clinicaltrials.gov). The study was approved by the National Medical Ethics Committee of the Republic of Slovenia. Written informed consent was obtained from all participants. All the procedures were performed in accordance with the Helsinki declaration. The CONSORT recommendations for reporting randomized trials were followed.

We enrolled 40 ASA (American Society of Anaesthesiologists) physical status I-III cooperative patients aged 18–80 years with a GSC (Glasgow coma score) of 15 who were scheduled for elective craniotomy for an intracerebral tumour. The patients included in the study were operated on by the same surgeon and anaesthetised by the same anaesthesiologist.

Patients were excluded if (a) they did not give written informed consent, (b) they had an endocrine system disease, (c) they were taking drugs that alter endocrine hormones (except dexamethasone that is invariably prescribed to all patients with brain tumours at this department), (d) they had a history of drug hypersensitivity, (e) they had a history of drug addiction, or (f) they received perioperative blood derivatives, such as red cell concentrates, platelet concentrates, fresh frozen plasma, cryoprecipitate, albumin, coagulation factors and immunoglobulins.

Using a computer-generated list the patients were randomised to either group by the third author IP, who was not involved in patient care. The first author (JMB) enrolled the patients and informed them about the participation in the study. The surgeon and the anaesthesiologist were blinded to the type of anaesthesia used.

All patients were on intravenous dexamethasone 4 × 4 mg/day^-1^ with the first dose given at least 1 day prior to surgery.

In the operating room standard monitoring was instituted. An arterial catheter was placed in the radial artery for continuous blood pressure monitoring. Advanced pulse contour cardiac output monitoring using the Vigileo/FloTrac device (Edwards Lifescience, CA, USA) was applied.

Patients were premedicated with midazolam (2-3 mg i.v.) and ondansetron (4–8 mg i.v.). Antibiotic prophylaxis with intravenous cefazolin 2 g/100 ml – 0.9 % NaCl was invariably used in all patients.

In propofol group anaesthesia was induced with propofol 1-2 mgkg^-1^ (Propoven, Fresenius Kabi AG, Bad Homburg, Germany) and in sevoflurane group with 6 % sevoflurane using a deep breath technique Sevorane, Abbott Laboratories, Texas, USA). Before intubation all patients received remifentanil 0.5–1 μgkg^-1^ (Ultiva, GlaxoSmithKline) and rocuronium 0.6 mgkg^-1^ (Esmeron, MSD, NY, USA).

After intubation both groups were ventilated mechanically with oxygen-air mixtures and with an I/E ratio of 1:2. The respiratory rate was adjusted to maintain normal values of paCO_2_ (partial pressure of carbon dioxide in arterial blood). The tidal volume was set to 8 mlkg^-1^. The peak inspiratory pressure was limited to 35 cm H_2_O. The fraction of inspired oxygen was adjusted to maintain normal values of partial pressure of oxygen in arterial blood. Anaesthesia was maintained by continuous infusion of propofol 4–6 mg kg^-1^h^-1^ in the propofol group and with sevoflurane 0.8–1 MAC in the sevoflurane group. Remifentanil was adjusted according to the degree of surgical manipulation (0.1–2 μg kg^-1^min^-1^) and increased when mean arterial pressure and heart rate increased by more than 30 % from baseline. The depth of anaesthesia was measured by the bispectral index (BIS) and the values were maintained at 40–60.

The following algorithm was used for haemodynamic management: intraoperative basal fluid replacement was realized with continuous infusion of 0.9 % NaCl 8 ml^-1^kg^-1^h^-1^ for the first hour, followed by 2.5 ml^-1^kg^-1^h^-1^. Additional boluses of 3 ml ^-1^kg^-1^ of colloid solution (Voluven 130/0.4 6 %; Fresenius Kabi AG, Bad Homburg, Germany) were given when stroke volume variation (SVV) measured by Vigileo/FloTrac system rose above 10 % (a sustained change during the previous 5 min) or in the case of a positive response (cardiac index [CI] increase above 10 %) to previous fluid challenge). If there was no improvement after fluid bolus, ephedrine (0.5 % Ephedrine, UMC Ljubljana Pharmacy, Slovenia) 5–10 mg (0.01 %, UMC Ljubljana Pharmacy, Slovenia) was instituted to maintain CI between 2 to 3.5 Lmin^-1^m^-2^. If CI > 2 Lmin^-1^m^-2^, SVV < 10 % and mean arterial pressure < 60 mmHg, phenylephrine 50–100 μg was given. If CI < 2 Lmin^-1^m^-2^, SVV < 10 % and heart beat < 40 min^-1^, atropine 0.5 mg was given. If mean arterial pressure increased by more than 30 % and heart rate by more than 30 % from baseline, the infusion of remifentanil was increased by 0.1 μg kg^-1^min^-1^. Any adverse haemodynamic events (an increase in mean arterial pressure of more than 30 % and/or in heart rate of more than 30 % from baseline) that did not respond to higher remifentanil infusion rate, were managed with urapidil or metoprolol, as appropriate. Blood loss was managed with colloids (Voluven 130/0.4 6 %; Fresenius Kabi AG, Bad Homburg, Germany) until a reduced PRBC transfusion trigger (haemoglobin level < 100 gl^-1^) was reached. Haemodynamic parameters were recorded continuously at 5-min intervals from the beginning of induction to discharge from the postanaesthesia care unit (PACU). During closing of the dura the patients were given a bolus dose of piritramide 0.1 mg/kg^-1^ (Dipidolor, Janssen-Cillag GmbH, Neuss, Germany).

We stopped sevoflurane and propofol infusion at the the last skin suture; remifentanil infusion was stopped after the removal of the Mayfield head holder.

Postoperatively patient-controlled analgesia (PCA) with continuous intravenous infusion of piritramide was started. The duration of the operation was defined as the time from the application of the Mayfield head holder to its removal. The duration of anaesthesia was defined as the time from induction to extubation. The time from the discontinuation of anaesthetics to tracheal extubation was also recorded. All the patients were extubated in the operating theatre and then transferred to the PACU.

The patients stayed in the PACU for not more than 2 h and were then taken to the intensive care unit (ICU) of the Department of Neurosurgery.

Standard postoperative monitoring generally used in these procedures was implemented. Oxygen was administered via a Venturi mask and titrated to the lowest level needed to achieve arterial oxygen saturation greater than 96 %. During the hospital stay the main investigator (JMB) visited the patients daily to check the adverse events and the given medication.

### Measurements

The data recorded included demographics, duration of surgery and anaesthesia, the consumption of intraoperative drugs, fluid balance, emergence and haemodynamic parameters. Postoperative complications were monitored during 15 days after surgery.

Postoperative complications were defined as any unintended changes in body function or well-being, such as hypertension (systolic blood pressure 30 % higher than the baseline level), postoperative nausea with vomiting, pain (visual analogue scale (VAS) > 3), infection, pulmonary, cardiovascular and neurological events, reoperation and death. The length of hospital stay was also recorded.

Serum levels of IL-6, IL-8 and IL-10 were measured in the pre-, peri- and postoperative periods. Arterial blood samples for determinations of cytokines IL-6, IL-8 and IL-10 were drawn at the following time points: (1) before induction, (2) during tumour resection, (3) at the end of surgery, (4) 24 h after surgery, and (5) 48 h after surgery.

For the analysis of serum IL-6, IL-8 and IL-10 blood samples were collected in tubes with no additive. After centrifugation serum samples were stored at –20 °C until analysis. The samples were analysed in a single batch. Chemiluminescent immunometric assay (Immulite analyzer; Siemens Healthcare, Erlangen, Germany) was used to measure the concentrations of IL-6, IL-8, and IL-10.

The primary outcome measures were serum levels of IL-6, IL-8 and IL-10.

The secondary outcome measures included the length of hospital stay and postoperative complications.

### Statistical analysis

The sample size was calculated from the previous pilot study of two independent groups (5 patients received propofol and 5 patients received sevoflurane) using a priori two tailed *t*-test power analysis. The difference in median serum concentration ratios (IL-10_i_/IL-10_i=1_) for each blood draw time point (*i* = 2, 3, 4, 5) against baseline IL-10_1_ concentration (before surgery and anaesthesia) was used for the effect size calculation and the resulting minimum sample size determination. For a significance level of 5 % (α = 0.05) and a power of 80 % (β = 0.2) the calculated minimum sample size was 19. To compensate for possible withdrawals, 20 patients were included into each group.

The two-tailed *t*-test with unequal variances or the Chi-square test were used to test the differences in demographic data, duration of the procedure and anaesthesia, drug consumption, fluid balance, postoperative complications, haemodynamic parameters and hospital stay. The exact Mann-Whitney *U*-test was used to compare the differences in IL-6_i_/IL-6_1_, IL-8_i_/IL-8_1_ and IL-10_i_/IL-10_1_ median ratio values and the changes in mean arterial pressure (MAP) and cardiac output (CO) during surgery between the groups.

The medians and the means of continuous variables are presented, and categorical data are summarized as counts. A *p*-value of less than 0.05 was considered statistically significant. Data were analysed by SPSS 13.0 software package.

## Results

**Fig. 1:**
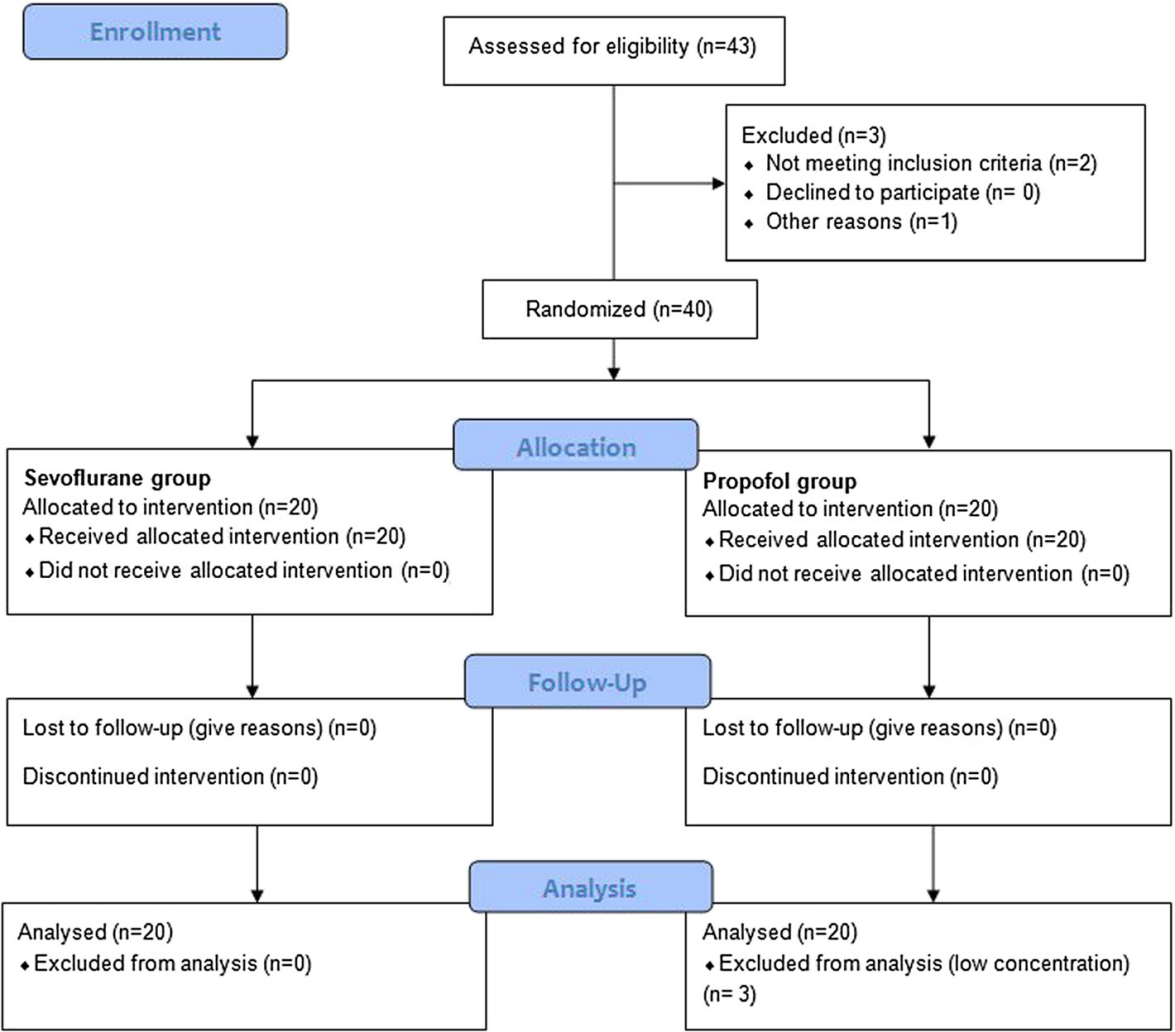
Flow diagram of the study

**Fig. 2:**
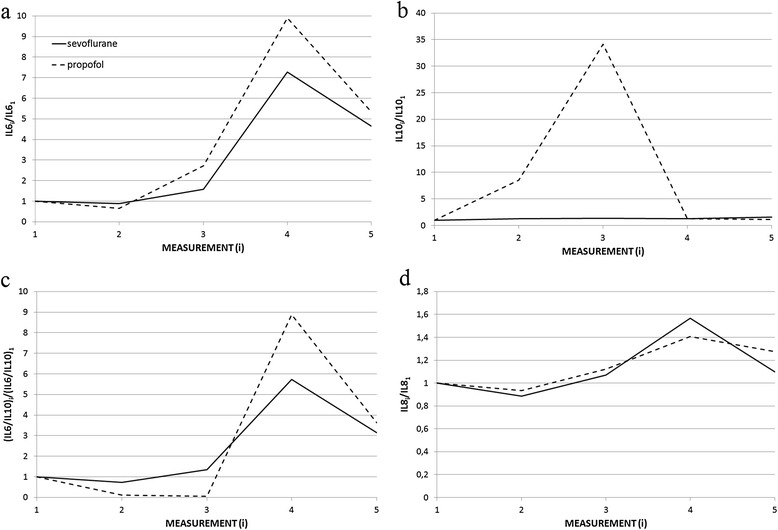
The ratios of IL-6_i_, IL-10_i_, (IL-6/IL-10)_i_ and IL-8_i_ to baseline values IL-6_1_, IL-10_1_, (IL-6/IL-10)_1_ and IL-8_1_ at various blood-sample draw time. Measurements (i): (1) before surgery and anaesthesia (2) during surgery, (3) at the end of surgery, (4) on the first postoperative day, (5) on the second postoperative day. The data are presented as the median values in all studied patients. **a** IL-6_i_/IL-6_1_ concentration ratio was not significantly different at any blood-sample draw time (measurement 2, *p* = 0.23; measurement 3, *p* = 0.12; measurement 4, *p* = 0.40; measurement 5, *p* = 0.55). **b** IL-10_i_/IL-10_1_ concentration ratio significantly increased in the propofol group at measurements 2 and 3 (measurement 2, *p* = 0.0001; measurement 3, *p* < 0.0001; measurement 4, *p* = 0.14; measurement 5, *p* = 0.38). **c** The (IL-6/IL-10)_i_/ (IL-6/IL-10)_1_ ratio in the propofol group significantly decreased at measurements 2 and 3 (measurement 2, *p* = 0.0001; measurement 3, *p* < 0.0001; measurement 4, *p* = 0.08; measurement 5, *p* = 0.29). **d** IL-8_i_/IL-8_1_ concentration ratio was not significantly different at any blood-sample draw time (measurement 2, *p* = 0.57; measurement 3, *p* = 0.67; measurement 4, *p* = 0.15; measurement 5, *p* = 0.43)

The study included 40 patients, 20 in the propofol group and 20 in the sevoflurane group (Fig. 1). None of the patients had signs of preoperative infection. All patients underwent craniotomy because of intracerebral tumour. Thirty-eight patients were operated on for the first time for the underlying pathology and 2 patients were reoperated. There were 37 supratentorial and and 3 infratentorial tumours (2 in the propofol group and 1 in the sevoflurane group). No significant differences were found between the groups regarding their demographics, underlying pathology and position during surgery (Table 1). Neither were there any significant differences in the length of hospital stay and intraoperative and postoperative variables that could have influenced the inflammatory response (Table 2). Patients who received propofol showed statistically significant increase in plasma IL-10 levels from baseline during surgery that lasted for 24 h after surgery (*p* = 0.0001) (Fig. 2b).

**Table 1:** Baseline demographics and surgical procedure

	S	P
N (number)	20	20
Age (years)	54 ± 14	53 ± 16
Weight (kg)	76 ± 12	79 ± 14
Gender (M/F)	6/14	10/10
ASA (I/II/III)	3/15/2	4/11/5
Tumour pathology		
Meningioma	10	10
Glioma	1	2
Glioblastoma	5	3
Astrocytoma	1	2
Pineal gland cyst	2	1
Other	1	2
Patient position		
Supine	14	15
Lateral	6	5
Duration of procedure (min)	163 ± 43	161 ± 68
Duration of anaesthesia (min)	182 ± 41	192 ± 71

The results are expressed as mean ± SD or number of patients

The differences between groups were not significant (*p* > 0.05)

*Abbreviations*: *ASA* American Society of Anaesthesiologists

**Table 2:** Intraoperative variables and postoperative complications

	S	P
Propofol (mg)		1060 ± 379
Remifentanil (mg)	11 ± 4	14 ± 9
Total loss of blood (ml)	405 ± 267	325 ± 226
Crystalloids (ml)	1065 ± 391	1176 ± 535
Colloids (ml)	441 ± 370	462 ± 327
Urine volume (ml)	750 ± 477	537 ± 440
Efedrin (mg)	7 ± 6	4 ± 6
Phenylephrine(mcg)	15 ± 37	30 ± 66
Intraoperative hypotension	5	4
Time to extubation (min)	9 ± 4	10 ± 3
Vomiting in PACU (yes/no)	7/13	3/17
VAS >3 in PACU (yes/no)	17/3	14/6
Additional piritramide (mg)	6 ± 4	4 ± 3
Postoperative hypertension	4	5
Vomiting in ICU (yes/no)	9/11	7/13
VAS >3 in ICU (yes/no)	1/19	0/20
Hospital stay (days)	12 ± 11	12 ± 8
Death	0	0
Reoperation	0	1
Postoperative CT of the head (good/edema/other)	18/1/1	16/2/2
Pulmonary complications		
(pulmonary embolism/other)	1/0	1/0
Infections (wound infection/other)	1/0	1/0
Cardiovascular complications	0	0
Neurological complications		
Seizures	0	2
Monoparesis	1	3
Balance disorder	1	2
Deafness or loss of smell	2	1
Confusion	1	0
Cerebrovascular insult	1	0

The results are expressed as mean ± SD or number of patients

The differences between groups were not significant (*p* > 0.05)

Patients in sevoflurane and propofol groups showed comparable perioperative changes in plasma levels of IL-6 (Fig. 2a), and IL-8 (Fig. 2d) at all time points. Patients who received propofol showed statistically significant decrease in IL-6/IL-10 ratio during surgery that lasted until the end of surgery (*p* = 0.0001) (Fig. 2c).

**Fig. 3:**
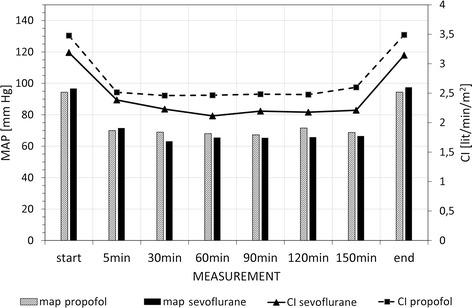
Changes in mean arterial pressure (MAP) and cardiac index (CI) during surgery. The data are presented as the mean values in all studied patients. The differences between the two groups were not statistically significant. (*p* - values for MAP during surgery from start to end: 0.49; 0.63; 0.07; 0.19; 0.17; 0.19; 0.25; 0.48). (*p* - values for CI during surgery from start to end: 0.13; 0.40, 0.09; 0.08; 0.09; 0.13; 0.12; 0.07)

The difference in mean arterial pressure (MAP) and cardiac output (CO) during surgery was not statistically significant between the groups (Fig. 3).

## Discussion

Our study of patients undergoing craniotomy for intracerebral tumours showed that propofol based anaesthesia was associated with significantly higher anti-inflammatory cytokine IL-10 levels than anaesthesia with sevoflurane, indicating that propofol anaesthesia may produce neuroprotective effects despite comparable neurological outcomes, length of hospital stay, and 15-day mortality rates of both groups.

Cytokines are a group of important inflammatory mediators that act in cascades, inducing or inhibiting each other [17]. They can enter the brain in many ways: they can cross the blood brain barrier (BBB) or bind to receptors associated with peripheral afferent nerves as part of the vagus nerve. They are produced in the CNS by activated microglia that have migrated as phagocytes, as well as by astrocytes and neurons [18, 19].

Finally, cortisol passes the blood brain barrier and influences the immune system in the CNS and peripheral nervous system [20]. In the present study we did not measure changes in cortisol levels. Citerio et al. showed, however, that during elective craniotomy intravenous anaesthesia was associated with a significant attenuation of neuroendocrine stress response [8]. A significant decrease in immune cell populations was found after intravenous induction in patients undergoing craniotomy [12].

Propofol reduces production of proinflammatory cytokines, alters expression of nitric oxide, and inhibits neutrophil function [21]. A recent in-vitro study showed that propofol almost completely inhibits lipopolysaccharide-induced activation of microglia and the production of proinflammatory cytokines [22]. It has been shown to attenuate neutrophil-mediated inflammatory diseases by blocking formyl peptide receptor 1 (FPR1) [23].

Our results suggest that TIVA with propofol exerts anti-inflammatory effects during and at the end of craniotomy, as reflected by a statistically significant decrease in IL-6/IL-10 ratio. These effects, however, seem to be only temporary, as IL-10 levels returned to baseline values on the first and second postoperative days. Sevoflurane had no major impact on IL-10 levels during either preoperative, perioperative or postoperative periods. In the postoperative period both anaesthestics showed proinflammatory action, as demonstrated by increased IL-6 levels, but the difference between the groups was not statistically significant. Neither anaesthetic had any major impact on the rate of postoperative complications. This finding suggests a potential medically important anti-inflammatory influence of propofol, which, however, should be confirmed by further studies.

Meta-analysis of several studies comparing propofol and volatile agents used for anaesthesia during elective craniotomy revealed no significant difference between both anaesthetic techniques in the majority of the measured outcomes [24]. According to Tange et al, who found increased cerebrospinal fluid levels of IL6 in the sevoflurane group, differences in neuroinflammatory response may be attributed to different anaesthetic techniques used [25].

In our study the sevoflurane and the propofol groups showed practically equal minor changes in IL-8 concentrations during and after surgery. The same results were found in patients undergoing craniotomy under general anaesthesia and those undergoing awake craniotomy [26]. IL-8 is an important proinflammatory inteleukin that may contribute to psychiatric complications of surgery [27].

Deviations of cytokine concentrations from the normal may be attributed to the effects of pre-existing medical illness, treatment modality, type of surgery or postoperative complications [18]. During neurosurgery neuroinflammation is caused by brain injury that is induced by various factors (brain tissue and vasculature manipulation, global haemodynamic changes) and affects normal brain structures [1].

Appropriate management of systemic and cerebral haemodynamic variables (cardiac output, arterial blood pressure, cardiac rhythm, cerebral blood flow) is a cornerstone of neuroanaesthesia [1]. In our study there were no significant differences in the degree of haemodynamic stability between the two groups (Fig. 3).

Corticosteroids are usually indicated in any brain tumour patient with symptomatic peritumoral oedema [28, 29]. Dexamethasone is generally used as it has relatively little mineralocorticoid activity, and is possibly associated with a lower risk of infection and cognitive impairment than other corticosteroids [28, 29]. At the Ljubljana Department of Neurosurgery a regimen with dexamethasone is invariably prescribed to all patients with brain tumours. This policy consitutes an inevitable limitation to our study as the impact of dexamethasone on the inflammatory system is well-known [28–30]. In their study, El Azab et al observed elevated IL -10 levels and decreased IL-6 and IL-8 levels in patients given dexamethasone in comparison to controls [30]. All patients included in our study were on the same dexamethasone regimen of 4 × 4 mg/day^-1^ for the same period of time before and after surgery. Because both groups were treated with dexamethasone according to the same protocol, we believe that the difference in cytokine profile changes is attributable to different anaesthetic techniques used.

Pain is another factor enhancing systemic inflammatory response and increasing serum cytokine levels [31]. In our study, possible impact of pain-related stress on the inflammatory response can be excluded. The difference in the amount of intraoperative remifentanil between the groups was not significant. The differences in VAS scores and requirements for additional analgesia with piritramide between the groups were not significant (Table 2).

The major advantage of our study is that the observation period was extended to 48 h postoperatively. Postoperative results failed to show clinical advantage of one anaesthetic technique over the other. However, we did not assess postoperative cognitive functions, long-term neurological morbidity and mortality, and quality of life. A longer observation period would be needed to clarify possible long-lasting effects of anaesthetics on neuroinflammation after craniotomy for intracerebral tumours.

## Conclusions

In conclusion, significant differences in cytokine profiles were found between the two anaesthesia groups. With propofol anaesthesia the concentration of anti-inflammatory cytokine IL-10 significantly increased during surgery. These findings, however, seem to have little effect on outcome, since neither sevoflurane nor propofol had any significant impact on the occurrence of postoperative complications. Our findings should incite future studies to prove potential medically important anti-inflammatory effects of propofol in neuroanaesthesia.
